# Precision fermentation of κ-casein using *Pichia pastoris*

**DOI:** 10.3389/fbioe.2026.1946745

**Published:** 2026-10-22

**Authors:** Laura Iaria, Nynke Flipse, Etske Bijl, Kasper Hettinga, Marc W. T. Werten

**Affiliations:** 1 Food Quality and Design Group, Wageningen University, Wageningen, Netherlands; 2 Wageningen Food and Biobased Research, Wageningen, Netherlands

**Keywords:** dairy proteins, O-glycosylation, Pichia pastoris, precision fermentation, protein secretion, proteolysis, recombinant protein, κ-casein

## Abstract

The environmental burden associated with conventional cheese production has driven interest in sustainable alternatives to animal-derived dairy proteins, including recombinant animal-free caseins produced via precision fermentation. Among caseins, κ-casein is essential for the renneting step during cheese production, making its recombinant production particularly relevant. Efficient secretion of the recombinant protein is key to economic feasibility at commercial scale, as it simplifies downstream processing and thereby reduces production costs. Here, we investigated the production and secretion of bovine κ-casein in the yeast *Pichia pastoris*. Successful secretion was achieved, and multicopy strains gave higher titers. However, considerable protein degradation was observed, also in bioreactor cultures. A yapsin 1 knockout and a yapsin1/protease A double knockout did not fully mitigate this, indicating the involvement of additional proteolytic activities. Moreover, κ-casein was detected in the periplasmic fraction of some of the strains tested, indicating that part of the recombinant protein was not released into the culture medium. The recombinant protein displayed heterogeneous electrophoretic behavior due to extensive *O*-linked glycosylation. Cytoplasmic expression of κ-casein was attempted as an alternative to secretory production, but did not result in high yields or clearly improved protein integrity. These results highlight the intrinsic challenges associated with κ-casein production in *P. pastoris*, where limited secretion efficiency, persistent degradation, and extensive *O*-glycosylation constrain yield and lead to protein heterogeneity. Overcoming these barriers will be essential to enable economically viable production of κ-casein through precision fermentation using this host.

## 1 Introduction

Precision fermentation uses genetically engineered microbial hosts to produce specific food ingredients, including proteins molecularly identical in sequence, structure, and functionality to their animal-derived counterparts. In the context of the protein transition, it has gained significant attention as a strategy to reduce reliance on conventional animal agriculture while potentially lowering environmental impact and addressing animal welfare concerns. Milk proteins represent a relevant and commercially attractive target, as dairy products are widely consumed, yet are associated with substantial environmental burdens (Poore and Nemecek, 2018). Among these proteins, caseins are of particular interest, as they are the main proteins in cheese. Caseins occur in four main types (α_s1_-, α_s2_-, β-, and κ-casein). Their ability to self-assemble into casein micelles mediated by the interaction of phosphorylated caseins with colloidal calcium phosphate is fundamental both to the nutritional role of milk, enabling the transport of calcium and phosphate, and to the structure and texture of cheese and other dairy products (Holt et al., 2013).

The α_s1_-, α_s2_-, and β-caseins constitute the bulk of the micelle and form its internal protein matrix through interactions with colloidal calcium phosphate via their phosphorylated residues. κ-Casein contains only a single phosphorylation site and is considered calcium-insensitive (Bijl et al., 2020). It is predominantly located at the micelle surface, where it is responsible for maintaining colloidal stability (Holt et al., 2013). κ-Casein is heterogeneously *O*-glycosylated within its C-terminal hydrophilic caseinomacropeptide region, with approximately 60% of the molecules carrying at least one glycan (Vreeman et al., 1986). This post-translational modification influences its physicochemical properties, including solubility and steric stabilization of the casein micelle (Bijl et al., 2020). During renneting in cheese production, added chymosin cleaves κ-casein at Phe105-Met106, releasing the caseinomacropeptide. The removal of this fragment colloidally destabilizes the casein micelle, triggering gelation, thereby leading to cheese curd formation (Holt et al., 2013).

Precision fermentation-based production of κ-casein thus targets the protein central to the colloidal stability of the casein micelle and key functionalities in cheese making. Understanding how differences in post-translational modifications relative to those in native κ-casein affect the functionality of the recombinant protein is essential for evaluating its suitability for dairy applications.

The yeast *P. pastoris* (*Komagataella phaffii*) is a well-established expression host in precision fermentation, being highly amenable to industrial fed-batch cultivation and having the machinery for performing eukaryotic post-translational modifications (PTMs) (Cereghino and Cregg, 2000). Its ability to, in principle, secrete the recombinant product represents a key advantage in precision fermentation, as extracellular accumulation eliminates the need for cell disruption and thereby simplifies downstream processing. Still, expression of caseins using *Pichia pastoris* has proven challenging. Attempts to produce β-casein in *P. pastoris* either failed to achieve secretion (Choi and Jimenez-Flores, 1996; Choi and Jiménez-Flores, 2001), or resulted in extensive degradation (Navone et al., 2023).

Here, we report the secretory production and characterization of κ-casein in *P. pastoris*, including an analysis of its posttranslational modifications.

## 2 Materials and methods

### 2.1 Media composition

Yeast extract Peptone Dextrose medium (YPD) (Duchefa) consisted of 10 g/L yeast extract, 20 g/L peptone and 20 g/L dextrose. For transformation, YPDS (YPD + sorbitol) plates were used, consisting of YPD medium supplemented with 1 M sorbitol and 20 g/L agar. Minimal Dextrose (MD) medium consisted of 13.4 g/L yeast nitrogen base without amino acids (Sigma), 20 g/L dextrose, and 0.4 mg/L biotin.

Basal salt medium with PTM1 trace elements was prepared as described by Zhang et al. (2000).

Buffered Minimal Glycerol medium (BMG) and Buffered Minimal Methanol medium (BMM) were prepared according to the Invitrogen EasySelect Pichia Expression system manual, with the exception that potassium phosphate buffer from the original protocol was replaced by 41.1 mM Na_2_HPO_4_ and 82.2 mM citric acid of medium to reach a pH of 3 (Prins and Billerbeck, 2021), and daily doses of 2% methanol were added for induction.

### 2.2 Plasmid and strain construction

*Pichia pastoris* strain X-33 was obtained from Invitrogen. The *P. pastoris yps1* strain was derived from GS115 (Invitrogen) by gene disruption in a previous study (Werten and de Wolf, 2005). The OPENPichia (NCYC 2543 *hoc1* ^*tr*^) *pep4 yps1* strain was obtained from VIB Ghent, Belgium (Claes et al., 2024).

Plasmids pPIC9K and pPICZ A were obtained from Invitrogen. Vector pPICZ9 was *de novo* synthesized (BaseGene) to be essentially identical to Invitrogen vector pPICZα A, except for replacement of the multiple cloning site sequence (from the first *Xho*I site to the stop codon of the His-tag) with that of Invitrogen vector pPIC9 (from *Xho*I to the stop codon).

The strains constructed and used in this study are summarized in Table 1. The amino acid sequence of bovine κ-casein A (kA) was obtained from UniProt (P02668) and modified by removing the native signal peptide (residues Met1-Ala21). The corresponding DNA sequence was codon-optimized for *P. pastoris* and synthesized by Genscript. The gene was supplied in vector pUC57, flanked by *Xho*I and *Eco*RI restriction sites for cloning into pPICZ9, or by *Psi*I and *Eco*RI sites for cloning into pPIC9K. For both vectors, this resulted in a seamless fusion of the encoded α-mating factor (α-MF) prepro secretory leader, the commonly used (Glu-Ala)_2_ spacer, and kA. In some constructs, an 8×His tag was added either to the N-terminus (kA_N) or the C-terminus (kA_C) of the protein. For the C-terminally His-tagged variants, a glycine-serine dipeptide was inserted upstream of the His-tag as a spacer. For intracellular expression, the kA_N gene was subcloned from vector pPICZ9 into pPICZ A via *Bst*BI/*Xho*I restriction cloning, introducing the required restriction sites by site-directed mutagenesis of the 5′ and 3′ ends of the gene. Full protein sequences are available in Supplementary Table S1.

**TABLE 1:** Strains used in this study.

Strain	Parental *P*. *pastoris* strain	Expression vector	Selection	Features	Culture system
kA	X-33	pPICZ9	Zeo^R^	-	Flasks and bioreactor
kA_7	X-33	pPICZ9	Zeo^R^	∼7 copies	Flasks
kA_17	X-33	pPICZ9	Zeo^R^	∼17 copies	Flasks and bioreactor
kA_N	X-33	pPICZ9	Zeo^R^	N-terminal His-tag	Bioreactor
kA_C	X-33	pPICZ9	Zeo^R^	C-terminal His-tag	Bioreactor
kA_Ny	GS115 *yps1* (Werten and de Wolf, 2005)	pPIC9K	His^+^	N-terminal His-tag	Bioreactor
kA_Cy	GS115 *yps1* (Werten and de Wolf, 2005)	pPIC9K	His^+^	C-terminal His-tag	Bioreactor
kA_Cyp	OPENPichia (NCYC 2543 *hoc1* ^*tr*^) *pep4 yps1* (Claes et al., 2024)	pPICZ9	Zeo^R^	C-terminal His-tag	Bioreactor
kA_Ni	X-33	pPICZ A	Zeo^R^	N-terminal His-tag	Bioreactor

Vectors were linearized with *Dra*I (pPICZ9 and pPICZ A) or *Sal*I (pPIC9K) before transformation to target integration at the desired locus (*AOX1* or *HIS4*, respectively).

### 2.3 *Pichia pastoris* transformation

*Pichia pastoris* cells were transformed by electroporation using a Gene Pulser (Bio-Rad) set to 1,500 V, 25 μF, and 400–800 Ω in 0.2-cm gap cuvettes. Transformants were subsequently plated on YPDS agar supplemented with 100 μg/mL Zeocin (for pPICZ9 and pPICZ A constructs) or on MD plates (for pPIC9K constructs) and incubated at 30 °C for 3–4 days. Individual colonies were selected for genotype confirmation by colony PCR (see Section 2.4), and Mut^+^ clones were chosen.

### 2.4 Colony PCR

Colonies were selected from each plate and cultured in ∼1 mL of YPD medium at 30 °C with shaking at 225 rpm. Aliquots of 10 μL from each culture were centrifuged for 5 min at 10,000 × g, and the supernatant was aspirated. The cell pellets were resuspended in 10 μL of Y-PER reagent (Thermo Scientific), vortexed for 10 s, and incubated under mild shaking at RT for 20 min. The lysates were then diluted 1:20 with sterile water and centrifuged as above. One μL of the supernatant was used as template for PCR amplification using DreamTaq Green PCR Master Mix (2X) (Thermo Scientific) with primers 5′AOX1 and 3′AOX1 (Invitrogen) at 0.5 μM and annealing at 57 °C.

### 2.5 Liquid post-transformational vector amplification

Liquid post-transformational vector amplification (LPTVA) was performed based on the method described by Aw and Polizzi (2016), using Zeocin selection with minor modifications. Briefly, strain kA_N (X-33, single-copy clone) was grown overnight in 5 mL of YPD medium supplemented with 100 μg/mL Zeocin. Every 24 h, a 10% (v/v) inoculum was transferred into fresh YPD medium containing progressively increasing concentrations of Zeocin (500, 1000, 1500, 2000, 2500, and 3000 μg/mL).

After 24 h of incubation in YPD containing 3000 μg/mL Zeocin, cultures were centrifuged, resuspended, and plated onto YPDS agar having the same antibiotic concentration. Single colonies were obtained by streak plating and subsequently grown in YPD without Zeocin. Cells were then harvested by centrifugation and stored for genomic DNA extraction and stock preparation.

### 2.6 Shake flask cultures

For each strain, 300 mL of BMG medium was inoculated in 1 L baffled flasks and grown over the weekend at 24 °C with agitation at 50 rpm. The optical density at 600 nm (OD_600_) was measured using a WPA CO 8000 spectrophotometer to calculate the volume required to inoculate 120 mL of BMM medium (pH 3) to a starting OD_600_ of 1.0. The appropriate volume of BMG culture was harvested by centrifugation for 5 min at 3,000 × g, resuspended in 5 mL of BMM, and subsequently used to inoculate 120 mL of BMM in 500 mL baffled flasks. Cultures were incubated at 30 °C with shaking at 225 rpm (New Brunswick Scientific Innova 4335). Methanol was added daily at 2% (v/v) of the initial culture volume.

### 2.7 Quantitative PCR

Quantitative PCR (qPCR) was used to determine the copy number of the expression cassette in clones obtained after LPTVA, as well as to assess copy-number stability during culturing. Genomic DNA (gDNA) was extracted using the YeaStar Genomic DNA Kit (Zymo Research) according to the chloroform-based protocol. DNA quality was evaluated by agarose electrophoresis, and DNA concentration was determined using the Quant-iT PicoGreen dsDNA assay (Invitrogen) according to the manufacturer’s instructions.

DNA samples were diluted to ∼40 pg/μL, corresponding to ∼200 pg of DNA per reaction. Genomic DNA from wild-type X-33 was used to generate a calibration curve (0, 8, 40, 200, 1000 and 5000 pg per well). *AOX1* copy number was determined using primer pairs targeting the *AOX1* promoter and the *ARG4* reference gene for normalization, following the approach described by Abad et al. (2010). qPCR reactions were done in an Applied Biosystems QuantStudio 1 real-time PCR system (Thermo Fisher Scientific) with PowerUp SYBR Green Master Mix (Thermo Fisher Scientific) under the following cycling conditions: 95 °C for 2 min, followed by 40 cycles of 95 °C for 15 s and 60 °C for 60 s. ROX was used as passive reference dye in all reactions. Melting curve analysis was done to confirm specific amplification. Copy numbers were determined using the relative standard curve method of the QuantStudio Design and Analysis software (v1.5.2.).

### 2.8 Bioreactor cultures

Fed-batch fermentations were performed according to Werten et al. (2009), as follows. The initial glycerol batch phase (40 g/L glycerol) was continued until glycerol depletion (typically ∼22 h). This was followed by a 1-h glycerol fed-batch phase at a constant feed rate of 18 mL of 50% (w/v) glycerol per L of initial culture volume per hour. During the subsequent transition to methanol, the glycerol feed was linearly decreased to 0 over 3 h. During this transition phase and the subsequent methanol fed-batch phase, methanol levels in the broth were controlled to ∼0.2% (w/v) using an off-gas sensor-controller. Methanol induction was maintained for 2–3 days. The DO setpoint was 50% during the glycerol batch phase, and 20% during the glycerol and methanol fed-batch phases. The pH was controlled at 3.0 throughout the fermentation by addition of ammonium hydroxide. Fermentations were tentatively conducted at pH 3 based on prior work with β-casein (to be published), where this pH resulted in higher titers than pH 6. After completion of the fermentation, cells were separated from the culture broth by centrifugation at 16,000 × g for 30 min at 20 °C using an RC-6 Plus centrifuge with SLA-3000 rotor (Sorvall). The resulting supernatant was clarified by microfiltration through Pall AcroPak 200 filters (0.8/0.2 µm).

### 2.9 SDS-PAGE, N-terminal sequencing and Western blotting

Sodium dodecyl sulfate-polyacrylamide gel electrophoresis (SDS-PAGE) was performed using the NuPAGE system (Invitrogen) with either 10% or 4%–12% Bis-Tris gels. Samples were prepared using NuPAGE LDS Sample Buffer (4X) and NuPAGE Sample Reducing Agent (10X) and separated using NuPAGE morpholineethanesulfonic acid (MES) SDS running buffer (20X). SeeBlue Plus 2 Pre-stained Protein Standard (Invitrogen) and PageRuler Plus Prestained Protein Ladder were used as markers. Electrophoresis was carried out for 40 min at 200 V. Following separation, gels were either stained or transferred to membranes for N-terminal sequencing or Western blot analysis.

Protein staining was performed using Coomassie SimplyBlue SafeStain (Invitrogen) according to the manufacturer’s instructions. For glycoprotein PAS staining, gels were incubated sequentially for 1 h in 12.5% (w/v) trichloroacetic acid (TCA), 1 h in 1% periodic acid and 3% acetic acid, and 1 h in 15% acetic acid, with the latter solution being replaced every 10 min. Gels were subsequently incubated with Schiff’s fuchsin-sulfite (Sigma S5133) reagent for 1 h at 4 °C in the dark.

For blotting, proteins were transferred onto polyvinylidene difluoride (PVDF) membranes (Invitrogen) pre-activated with ethanol. For N-terminal sequencing, membranes with a 0.2 μm pore size were used, and proteins were transferred using CAPS buffer (10 mM, pH 11) containing 10% (v/v) ethanol. For Western blot analysis, 0.45 μm pore size PVDF membranes were used, and protein transfer was performed using Tris-glycine buffer (25 mM Tris, 190 mM glycine). Blotting was at 100 V for 1 h in a Mini Trans-Blot Cell (Bio-Rad).

For N-terminal sequencing, membranes were briefly washed with Milli-Q water followed by 100% (v/v) ethanol for 10 s, and stained for 1 min with Coomassie Brilliant Blue (PhastGel Blue R; Amersham Pharmacia Biotech). The protein bands of interest were excised using a clean scalpel and submitted for N-terminal sequencing by Edman degradation (Iowa State University Protein Facility).

For Western blot analysis, membranes were blocked for 1 h using Pierce Protein-Free Blocking Buffer (Thermo Fisher Scientific) and incubated with primary antibodies diluted 1:5,000 in blocking buffer for 1 h. Membranes were then washed three times for 10 min with Tris-buffered saline containing 0.1% Tween 20 (TBS-T), followed by one wash with Tris-buffered saline (TBS). Secondary antibodies diluted 1:10,000 in blocking buffer were applied for 1 h, followed by three washes with TBS-T and one wash with TBS. The membrane was developed using Pierce ECL Western blotting Substrate (Thermo Scientific).

Primary antibodies used were either protein A-purified rabbit serum anti-κ-casein antibody (NewMilkLab) or a 6×His tag polyclonal antibody (Invitrogen). The secondary antibody was goat anti-rabbit IgG (H + L) conjugated to horseradish peroxidase (HRP) (Invitrogen) for chemiluminescent detection.

Gels and Western blots were imaged using the ChemiDoc MP Imaging System (Bio-Rad, Hercules, California, USA) with Image Lab 4.1 software (Bio-Rad). This software was also used to overlay the visible bands of prestained molecular weight markers with the chemiluminescent signal obtained from antibody detection. In some cases (SDS-PAGE gels only), imaging was performed using a GS-800 Calibrated Densitometer (Bio-Rad).

Band densitometry of Western blots was performed using ImageJ.JS (NIH, Bethesda, MD). Band areas were measured using the rectangle and wand tools, with background subtracted via the plot’s baseline. Protein quantities in samples were estimated relative to a single known amount of bovine κ-casein (NewMilkLab) included on the same blot.

### 2.10 Purification of his-tagged proteins

The pH of the microfiltered supernatant was adjusted to 8.0 with NaOH, and precipitated salts were removed by centrifugation at 2,000 × g for 10 min. The clarified supernatant was then subjected to gravity-flow Ni-NTA affinity chromatography at pH 7.4 using HisPur Ni-NTA Resin (Thermo Scientific) packed in PD-10 disposable columns (Cytiva). Purification under native conditions was performed according to the protocol of the manufacturer of the Ni-NTA resin. For denaturing purification, the *P*. *pastoris* broth was subjected to ultrafiltration/diafiltration using a 3 kDa molecular weight cut-off Amicon Ultra Centrifugal Filter (Millipore) to exchange the culture medium with equilibration buffer (20 mM sodium phosphate, 300 mM sodium chloride, 10 mM imidazole and 6 M guanidine hydrochloride, pH 8), and the resulting sample was subsequently used for purification. The same buffer was used throughout, but with 25 mM imidazole for washing and 250 mM imidazole for elution. For purifications performed under protease-inhibited conditions, cOmplete EDTA-free protease inhibitor cocktail (Roche) was added to all purification buffers at the manufacturer-recommended working concentration.

Following purification, eluates were subjected to ultrafiltration/diafiltration using a 3 kDa molecular weight cut-off centrifugal filter (Millipore Ultra-0.5) to exchange the buffer with TBS (20 mM Tris, 150 mM NaCl, pH 6.7) and to concentrate the protein.

### 2.11 Cellular and periplasmic protein extraction

For extraction of cellular proteins, *P*. *pastoris* cells were resuspended at 200 mg/mL wet weight in SED buffer (1 M sorbitol 1M, 25 mM EDTA, 40 mM DTT; Hartmann et al., 2017) supplemented with cOmplete protease inhibitor cocktail (Roche). Resuspended cells were lysed in a Precellys 24 Bead Mill Homogenizer (Bertin Technologies) using 0.5 mm Zirconia/Silica beads (BioSpec) at 2,500 rpm for 60 s over nine cycles, with 60 s cooling intervals between cycles. Periplasmic proteins were extracted using a zymolyase-based spheroplasting method, adapted from Hartmann et al. (2017). Briefly, 1 g (wet weight) of cells was washed sequentially in SED buffer and 1 M sorbitol, centrifuging for 5 min at 5000×g (4 °C) after each wash. Cells were then resuspended in citrate-glycerol buffer (20 mM trisodium citrate, 10% glycerol, pH 5,8 adjusted with HCl) supplemented with cOmplete protease inhibitor cocktail (Roche), and treated with Zymolyase 20 T (Amsbio, United Kingdom: 20 U per Gram of wet cells). After incubation at room temperature for 15 min under gentle agitation, spheroplasts were removed by centrifugation for 5 min at 750 × g (RT), using reduced acceleration and deceleration to prevent lysis. The supernatant, containing the periplasmic protein fraction, was collected for further analysis.

### 2.12 Alpha-mannosidase treatment

To remove possible *O*-glycans, purified protein samples were digested with Jack bean α(1–2,3,6)-mannosidase (ProZyme GKX-5010). Briefly, ∼2 μg of purified protein was incubated with -α-mannosidase (0.45 units) in the supplied reaction buffer, and the reaction was carried out at 37 °C for 18 h.

### 2.13 LC-MSMS

#### 2.13.1 Sample preparation

For in-gel digestion (IGD), SDS-PAGE gels stained using Coomassie dye (SimplyBlue SafeStain; Invitrogen) were rinsed thoroughly with water. Disulfide bonds were reduced with 50 mM ammonium bicarbonate containing 15 mM dithiothreitol (DTT) for 30 min at 45 °C. After washing with water, cysteines were alkylated with 20 mM iodoacetamide prepared in Tris buffer (pH 8.0) for 30 min at room temperature in the dark. Gels were washed extensively with water to remove residual reagents. Protein bands were excised, cut into ∼1 mm^3^ pieces, and transferred to low-binding microcentrifuge tubes. Gel pieces were incubated overnight at room temperature with sequencing grade trypsin (Roche, 5 ng/μL in 50 mM ammonium bicarbonate), ensuring complete coverage of the gel fragments. Peptides were extracted by acidifying the digestion mixture with trifluoroacetic acid (pH 2–4), followed by extraction with 50% acetonitrile containing 0.1% formic acid. Samples were dried in a vacuum concentrator and reconstituted in 0.1% formic acid prior to LC–MS/MS analysis.

#### 2.13.2 LC-MS/MS acquisition

Nano LC-MS/MS was performed as described by Zheng et al. (2024).

#### 2.13.3 Data analysis

Peptide identification and quantification were performed using MaxQuant (v 2.0.3.0, Cox and Mann, 2008; Sinitcyn et al., 2021). Spectra were searched against the UniProt *P. pastoris* GS115 reference proteome (UP000000314) supplemented with a database of common contaminants, including bovine trypsin (P00760). Unspecific enzyme search was performed to allow identification of peptides generated by endogenous *Pichia* proteases. Precursor and fragment mass tolerances were set to 4.5 ppm and 20 ppm, respectively. Cysteine carbamidomethylation was specified as a fixed modification. The following variable modifications were included in the search parameters: methionine oxidation; N-terminal acetylation; deamidation; acrylamide adduct formation; and phosphorylation of serine, threonine, and tyrosine residues. False discovery rates (FDR) for both peptide-spectrum matches and protein identifications were controlled at 1%. Modified peptides were subsequently filtered using a minimum score threshold of 65, a maximum peptide length of 25 amino acids, a localization probability of at least 0.5, and posterior error probability (PEP) values ≤ 0,01.

### 2.14 MALDI-TOF

Matrix-assisted laser desorption/ionization time-of-flight (MALDI-TOF) mass spectrometry was performed as described by Domeradzka et al. (2016) using a Bruker Autoflex maX LRF instrument. Samples were desalted prior to analysis using Micro Bio-Spin 6 kDa cut-off size exclusion chromatography columns (Bio-Rad).

## 3 Results and discussion

### 3.1 Shake-flask cultures of κ-casein strains with different copy numbers

For secretory production, the gene for κ-casein (kA) without its signal peptide was fused to the α-mating factor prepro leader, retaining the EAEA (Glu-Ala) spacer residues to ensure steric accessibility for Kex2-mediated maturation of the secreted protein. The gene was cloned into plasmid pPICZ9 and used to transform *P. pastoris* strain X-33, generating the single copy strain kA. LPTVA (Aw and Polizzi, 2016) was used to derive multicopy clones of strain kA, to assess the effect of copy number on recombinant κ-casein yield. Following selection, two clones were chosen for further analysis: a ∼7-copy clone (kA_7) and a ∼17-copy clone (kA_17), which were compared to the original single-copy kA strain. Shake-flask cultures at pH 3 were performed with the three κ-casein-producing clones (kA, kA_17, kA_7). The parental strain X-33 was included as a negative control. The stability of gene copy number in clones kA_7 and kA_17 was evaluated by qPCR using genomic DNA samples collected at the start and at the end of the culture. The analysis indicated no clear copy loss in either strain (Supplementary Figure S1A).

Western blot analysis of the cell-free broth (Figure 1) detected secreted κ-casein in all producing strains (the original image of this, and all other Western blots below, can be found in the Supplementary Materials S6–S15). The bands showed a smeared pattern at ∼38 kDa, with faint lower-molecular-weight bands likely representing degradation products. The theoretical molecular mass of kA variant A is 18,974 Da; however, extensive glycosylation can substantially increase the apparent molecular weight of proteins in SDS-PAGE, and typically results in a smeared migration pattern due to the presence of multiple glycoforms. Also in the bovine κ-casein control, known to be glycosylated, the major band migrated at ∼28 kDa rather than at the theorical molecular weight (Figure 1). The smeared signal between 49 and 62 kDa is likely attributable to a co-purified milk protein that cross-reacts with the polyclonal anti-κ-casein antibody.

**FIGURE 1:**
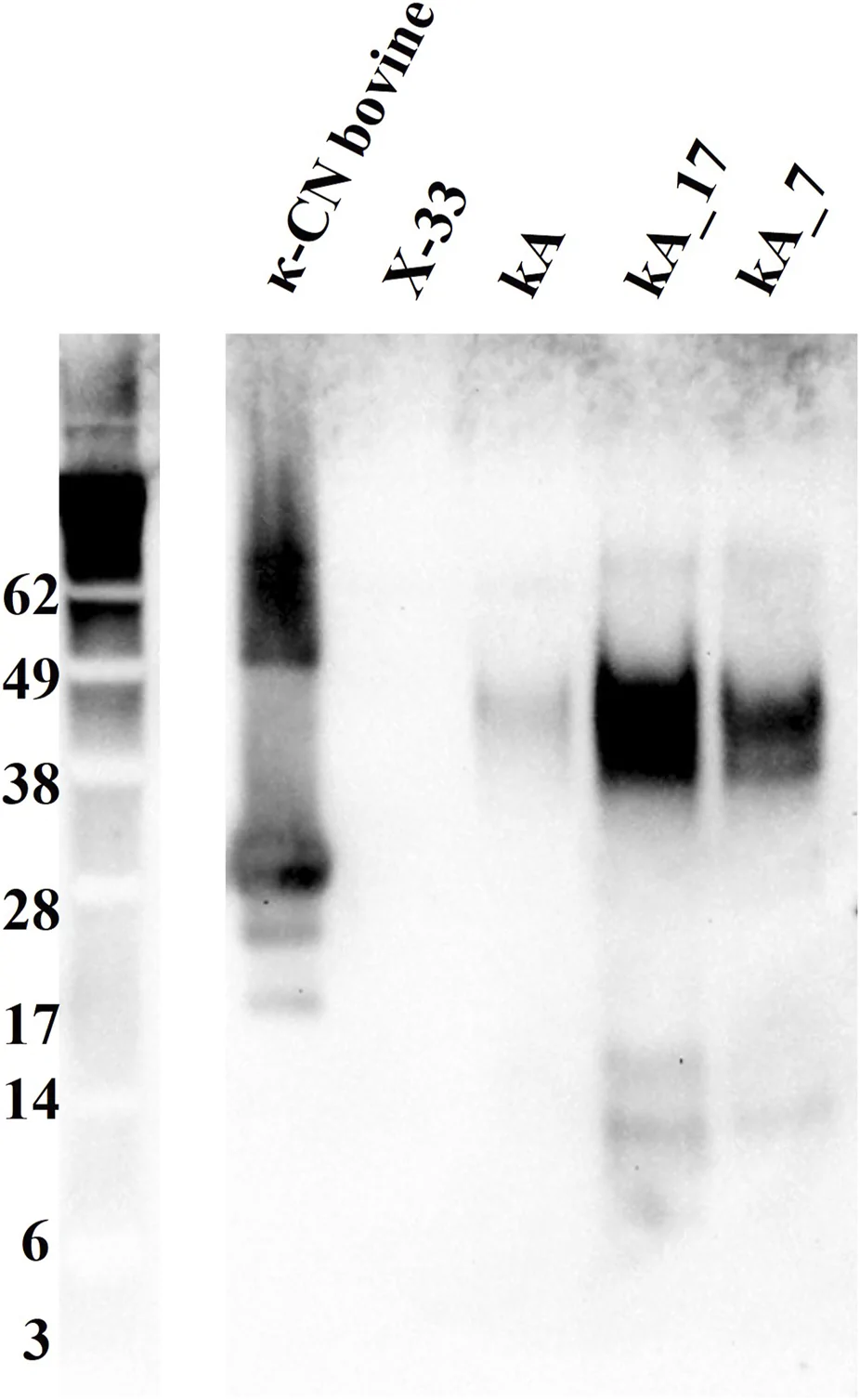
Western blot of fermentation supernatants from shake-flask cultures of κ-casein-producing *Pichia pastoris* clones with different construct copy numbers, probed with anti-κ-casein primary antibody. Proteins were separated by SDS-PAGE (10% acrylamide), with equal volumes (19 µL) of fermentation supernatant loaded per lane. Wild-type strain X-33 was included as negative control. For reference, 2 µg of bovine κ-casein was loaded.

The titers of recombinant κA casein were estimated by densitometry (Supplementary Figure S2) using a single bovine κ-casein reference standard, resulting in values in the cell-free broth of 28 mg/L for kA, 89 mg/L for kA_7, and 133 mg/L for kA_17. These are calculated values only and not intended to imply a corresponding level of precision; they merely serve to provide rough estimates. While the observed trend is consistent with a gene dosage effect, genomic position effects cannot be excluded, as the integration sites have not been characterized.

### 3.2 Bioreactor fermentation

To assess production of κ-casein under more industrially relevant conditions, the single-copy kA strain was cultivated in methanol fed-batch mode in a benchtop bioreactor at pH 3. The durations of the batch and methanol induction phases and the final wet cell weight for this fermentation, as well as for all other fermentations below, are reported in Supplementary Table S3. The supernatant at the end of the fermentation was analyzed by SDS-PAGE followed by Coomassie staining. However, we did not observe any potential casein-related bands distinct from the typical host-derived protein profile. A His-tag (8×His) was therefore subsequently introduced to enable straightforward purification and further characterization of the protein. The resulting single-copy strains, kA_N (N-terminally His-tagged κ-casein), and kA_C (C-terminally His-tagged κ-casein) were fermented in benchtop bioreactors as before. Casein expression was assessed using a Western blot, probed with either an anti-κ-casein (Figure 2A) or an anti-His-tag primary antibody (Figure 2B).

**FIGURE 2:**
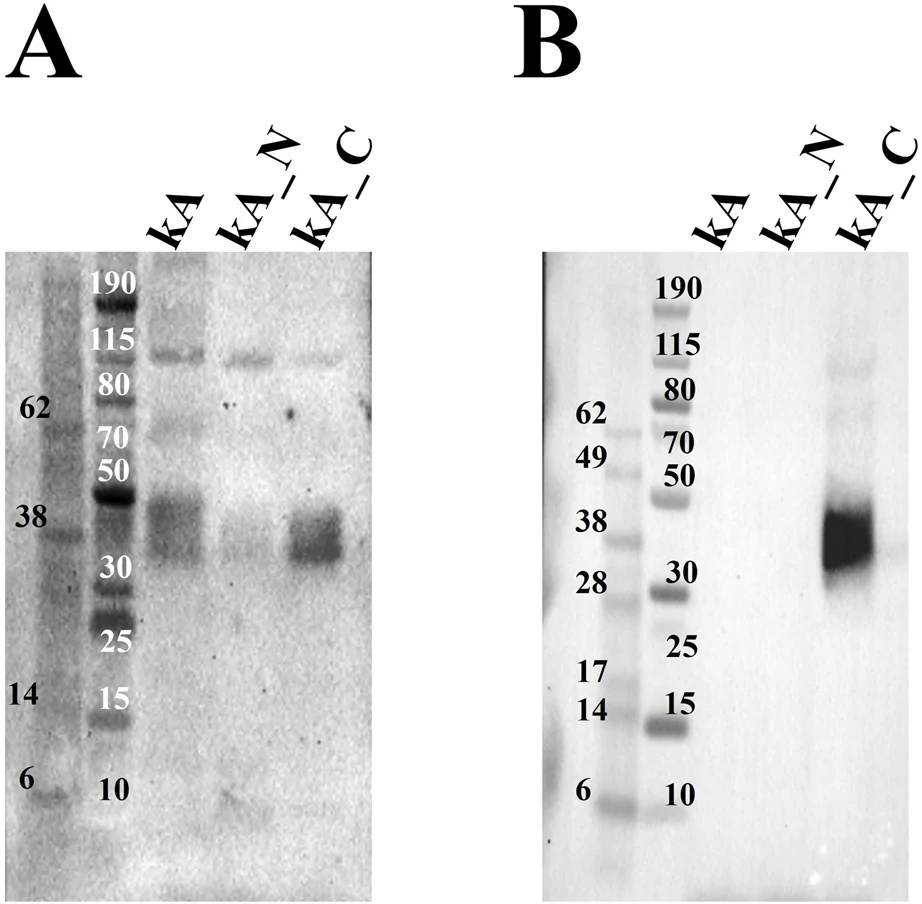
Western blot of fermentation supernatants from bioreactor cultures of κ-casein-producing *Pichia pastoris* strains probed with **(A)** anti-κ-casein primary antibody, and **(B)** anti-His-tag primary antibody. Proteins were separated by SDS-PAGE (4%–12% acrylamide). Equal volumes (19 µL) of fermentation supernatant were loaded per lane.

The band corresponding to κ-casein in Figure 2A appeared as a diffuse smear with an apparent molecular weight of ∼38 kDa, similar to what was observed in the shake-flask experiments and possibly representing the glycosylated protein. As expected, no signal was detected for strain kA in the anti-His-tag Western blot (Figure 2B), as this construct does not contain a His-tag. Whereas no band was observed for kA_N, kA_C did show a clear immunoreactive band. This might be due to the lower titer of kA_N compared to kA_C, as evident from the Western blot with anti-kappa antibody (Figure 2A), although the signal ratios argue against this as sole explanation. Another possible explanation for the lack of detection of kA_N by anti-His-tag antibody could be proteolytic cleavage near the N-terminus, which would result in the release of a small tag-bearing fragment that may escape detection by Western blot. An additional weak band appears in Figure 2A at ∼115 kDa for all three protein variants, and for kA also at ∼70 kDa, presumably attributable to non-specific binding to host proteins.

For further characterization of the produced casein, the His-tagged variants kA_N and kA_C were purified by Ni-NTA affinity chromatography. After purification under denaturing conditions, the protein was subjected to buffer exchange using 3 kDa ultrafiltration/diafiltration to remove imidazole and to concentrate the protein. It was then analyzed by SDS-PAGE (Figure 3A). kA_N appears predominantly as a major band migrating at approximately 6 kDa (band 1), together with two slightly slower-migrating bands (bands 2 and 3), and an additional fuzzy band at ∼38 kDa (band 4). The low molecular weight bands suggest extensive proteolytic degradation of the protein. For kA_C, two major bands are observed (band 5 at ∼28 kDa, and band 6 at ∼38 kDa). The pattern observed after purification was not fully consistent with the Western blot of the cell-free broth (Figures 2A,B). First, the low-molecular-weight bands 1–3 were observed by Coomassie staining of purified kA_N, but were not detected in the anti-casein and anti-His-tag Western blots. These bands may correspond to truncated fragments that either lack the epitope recognized by the primary antibody or escape detection by passing through the membrane during blotting. Second, while two distinct bands at ∼28 and ∼38 kDa were observed in the purified kA_C sample with Coomassie staining, the Western blot showed two poorly resolved bands migrating at around ∼38 kDa (anti-casein; Figure 2A) or a single blurry band at that position (anti-His-tag; Figure 2B).

**FIGURE 3:**
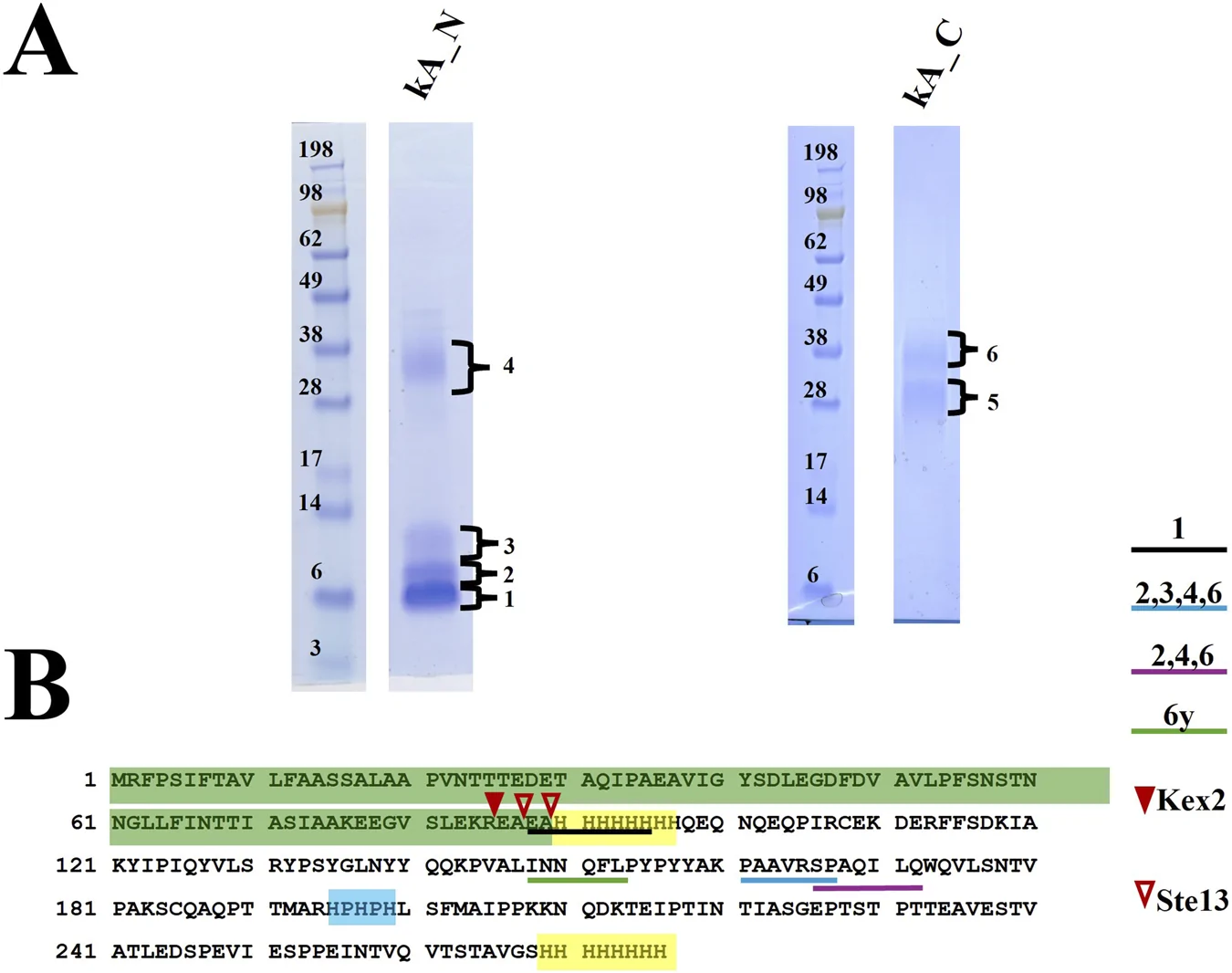
**(A)** SDS-PAGE (10% acrylamide) of kA_N and kA_C purified from the cell-free broth of bioreactor cultures by Ni-NTA chromatography. Brackets indicate the bands selected for N-terminal sequencing by Edman degradation. The amount of eluate loaded in each lane corresponds to ∼6 mL of cell-free culture broth. **(B)** Protein sequence of kA_N and kA_C. For brevity, His-tags are shown at both termini; however the tag is located at the N-terminus in kA_N and at the C-terminus in kA_C. Edman sequencing results are underlined with different colors, with numbers indicating the corresponding bands in the legend. The HPHPH sequence discussed in Section 3.2 is highlighted in light blue. The α-mating factor secretory leader is highlighted in green. Kex2 and Dipeptidyl Aminopeptidase A (Ste13) cleavage sites for proteolytic maturation are indicated in the sequence.

The numbered bands indicated in Figure 3A were subjected to N-terminal sequencing to confirm their identity as κ-casein. The sequencing results are displayed in Figure 3B (underlying data shown in Supplementary Table S4). Band 5 was not included in this analysis because, although it was visible in SDS-PAGE, it was consistently not detectable on the sequencing blot. For band 1, the identified sequence EAHHHHH matches the expected N-terminus of kA_N, extended with a single Glu-Ala dipeptide. The presence of this dipeptide indicates incomplete processing of the (Glu-Ala)_2_ spacer often placed between the α-MF leader and the protein of interest, as is commonly observed (Ahmad et al., 2014). The identified sequence is consistent with the purification strategy, as the His-tag would be expected to be present, given that the protein was purified via Ni-NTA affinity chromatography. Remarkably, in all other bands of purified kA_N, the His-tag was not detected. Instead, N-terminal sequencing revealed the internal sequences PAAVRSP and/or SPAQILQ (Figure 3B). Internal fragments without N-terminal His-tag might hypothetically result from proteolytic degradation during or directly after elution of the protein from the Ni-NTA resin. However, this possibility is practically excluded because repeating the purification in the presence of a broad spectrum protease inhibitor cocktail did not alter the band pattern in SDS-PAGE (Supplementary Material, Supplementary Figure S3). Instead, we attribute the copurification of internal fragments under medium stringency washing to the histidine-rich sequence stretch HPHPH in κ-casein. This sequence features two overlapping HXH motifs, which can each act as bidentate ligands in a preferred coordination geometry (Knecht et al., 2009), likely leading to relatively strong affinity. Although the effect of Pro as X has not been reported in the literature, its conformational restraint might further raise affinity owing to increased local cooperativity and reduced entropic penalty upon binding. The fact that N-terminal sequencing of kA_N bands 2 to 4 revealed internal sequences confirms the earlier interpretation of the anti-His-tag Western blot (Figure 2B) that the N-terminal His-tag in kA_N may have been proteolytically cleaved off. Although band 1, according to N-terminal sequencing, did retain the His-tag, it was not detected in either the anti-κ (Figure 2A) or anti-His-tag (Figure 2B) Western blot. As speculated above, this may be due to poor membrane transfer of low-molecular weight species.

For band 6 of kA_C, the N-terminal sequence identified corresponds to the internal sequences PAAVRSP and SPAQILQ. These are the same sequences as found for kA_N bands 2, 3 and 4, apparently resulting from cleavage C-terminal of basic residues at YYAK↓PAAV and AAVR↓SPAQ, respectively (Figure 3B). This might be caused by yapsin 1 (Yps1) protease; a GPI-anchored cell wall aspartyl protease that can cleave secreted and cell wall proteins. The protease is known to sometimes also be involved in endoproteolysis of secreted heterologous proteins in *P. pastoris* (Silva et al., 2011; Werten and de Wolf, 2005).

In view of the improved performance of strain kA_17 in shake-flask experiments (Section 3.1), we also evaluated it in a bioreactor culture. Relative expression levels were assessed by Western blot analysis using anti-κ antibody by comparison with protein produced in a single-copy reference strain (kA_N) (Figure 4). kA_17 exhibits a substantially higher signal intensity to the extent that single-copy kA_N is invisible in the same exposure. This large difference is similar to that observed in the Western blot of the shake-flask expression of multicopy versus single-copy expression (Figure 1), although under those conditions the protein in the single-copy strains is still just visible. The strong intensity of the main band indicates that protein production is substantially enhanced in kA_17. The stability of gene copy number was evaluated by qPCR using genomic DNA samples collected from the preculture flask immediately before bioreactor inoculation and at the end of the bioreactor culture. Also in this case, the analysis indicated no significant loss of copies (Supplementary Figure S1B).

**FIGURE 4:**
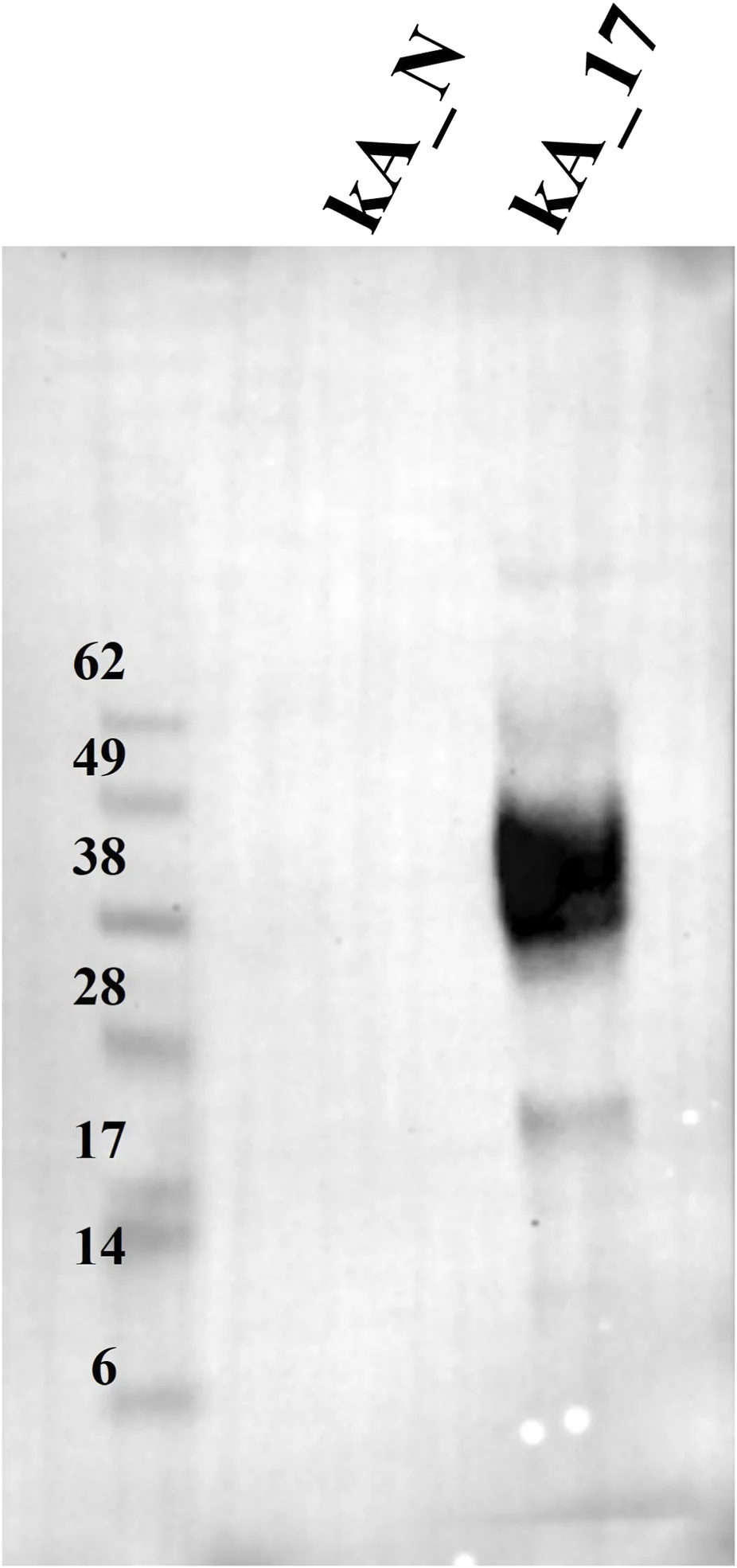
Western blot (anti-κ-casein primary antibody) of fermentation supernatants from bioreactor cultures of kA_N and kA_17 loaded at equal volumes (19 µL). Proteins were separated by SDS-PAGE (4%–12%).

### 3.3 Protease knockout strains

Since SDS-PAGE and N-terminal sequencing indicated proteolytic degradation of κ-casein, protease knockout strains were evaluated to assess the contribution of specific proteases and to improve the integrity of the recombinant protein. Because the observed cleavage immediately downstream of single Lys and Arg residues could match the sequence specificity of Yps1 protease, a *yps1* knockout strain was chosen for production of κ-casein with either an N-terminal (kA_Ny) or C-terminal His-tag (kA_Cy). The protein sequence of κ-casein contains a considerable number of hydrophobic dipeptides, which are a potential target for vacuolar Protease A (Dreyer, 1989). Because vacuolar proteases (Protease A, as well as Protease B and carboxypeptidase Y activated by protease A) released through cell lysis can contribute to extracellular degradation (Sinha et al., 2005), the C-terminally His-tagged κ-casein construct was additionally expressed in a *pep4 yps1* double knock-out strain (kA_Cyp). Figure 5 shows an anti-His-tag Western blot of bioreactor fermentation supernatants of C-terminally His-tagged κ-casein produced in the wild-type strain (kA_C) and in protease-deficient strains (kA_Cy and kA_Cyp). The results suggest a higher κ-casein yield in the *pep4 yps1* strain, but no clear differences in banding patterns.

**FIGURE 5:**
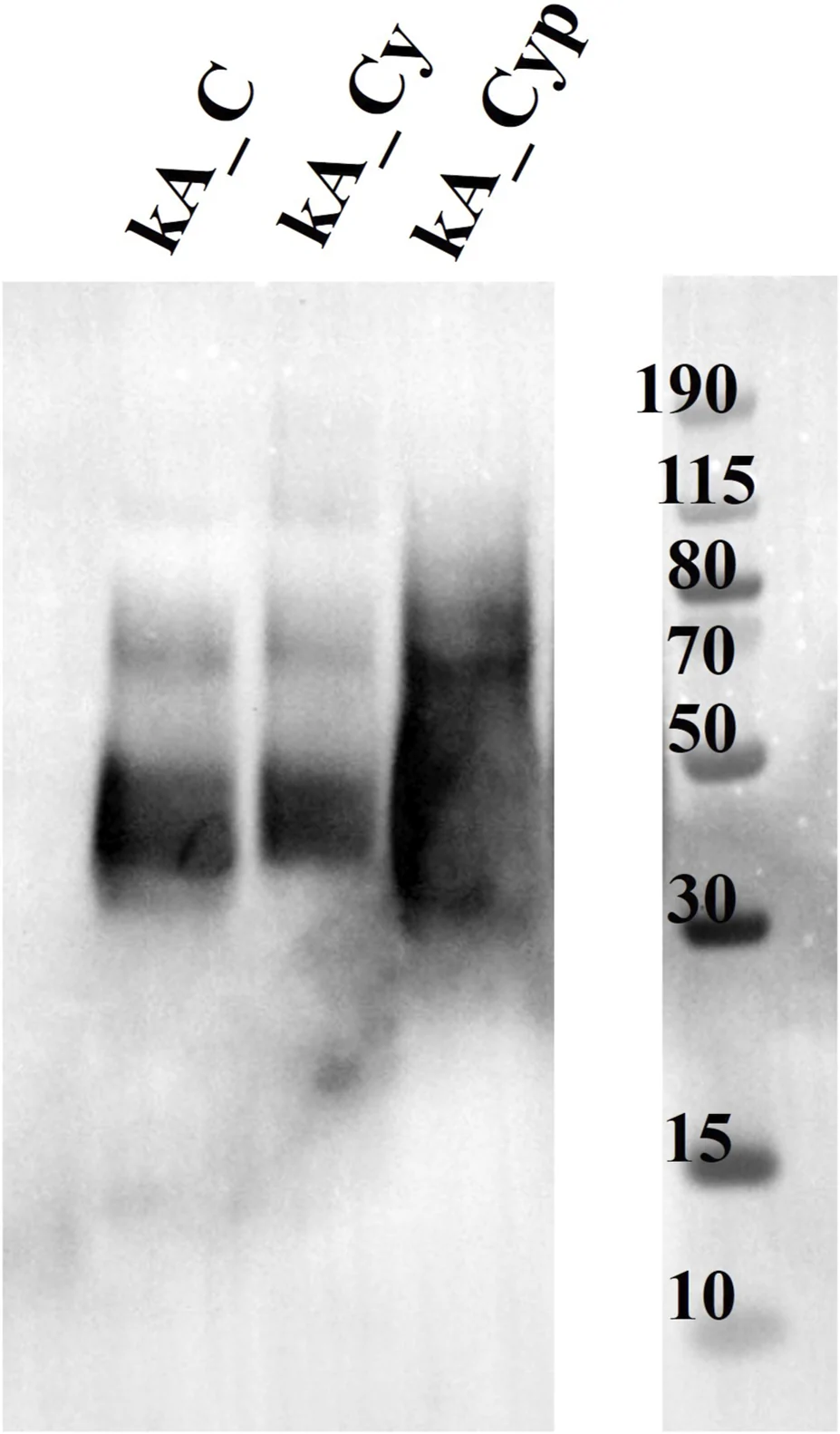
Western blot (anti-His-tag primary antibody) of fermentation supernatants from κ-casein-producing *Pichia pastoris* protease-knockouts versus wildtype in bioreactor cultures. Proteins were separated by SDS-PAGE (4%–12% acrylamide) and equal volumes of fermentation supernatant (19 µL) were loaded.

Ni-NTA affinity chromatography was used to allow further characterization of the proteins. Although denaturing conditions were used in Section 3.2, native conditions were used here because in preliminary trials we did not observe appreciable differences for *P. pastoris*-produced κ-casein. The similar behavior of the target protein under both conditions is consistent with its intrinsically disordered nature. The eluates were analyzed by SDS-PAGE (Figure 6A). The observed band pattern appears similar between the wild-type and *yps1* strains. However, N-terminal sequencing of band 6y (Figure 6A; Supplementary Table 10) of kA_Cy revealed the sequence INNQFL, which lies slightly upstream of the previously obtained sequences for band 6 (Figure 3B). This indicates that, although deletion of Yps1 protease did eliminate the previously observed cleavage events, κ-casein was still cleaved by another protease at an upstream site. The cleavage events identified for kA_N and kA_C in both the wild-type and *yps1* strains are summarized schematically in Figure 7. Note that the >30 kDa apparent molecular weight in SDS-PAGE of some of the bands corresponding to the ≤12 kDa part of the molecule downstream of the yapsin 1 cleavage site suggests very extensive *O*-glycosylation.

**FIGURE 6:**
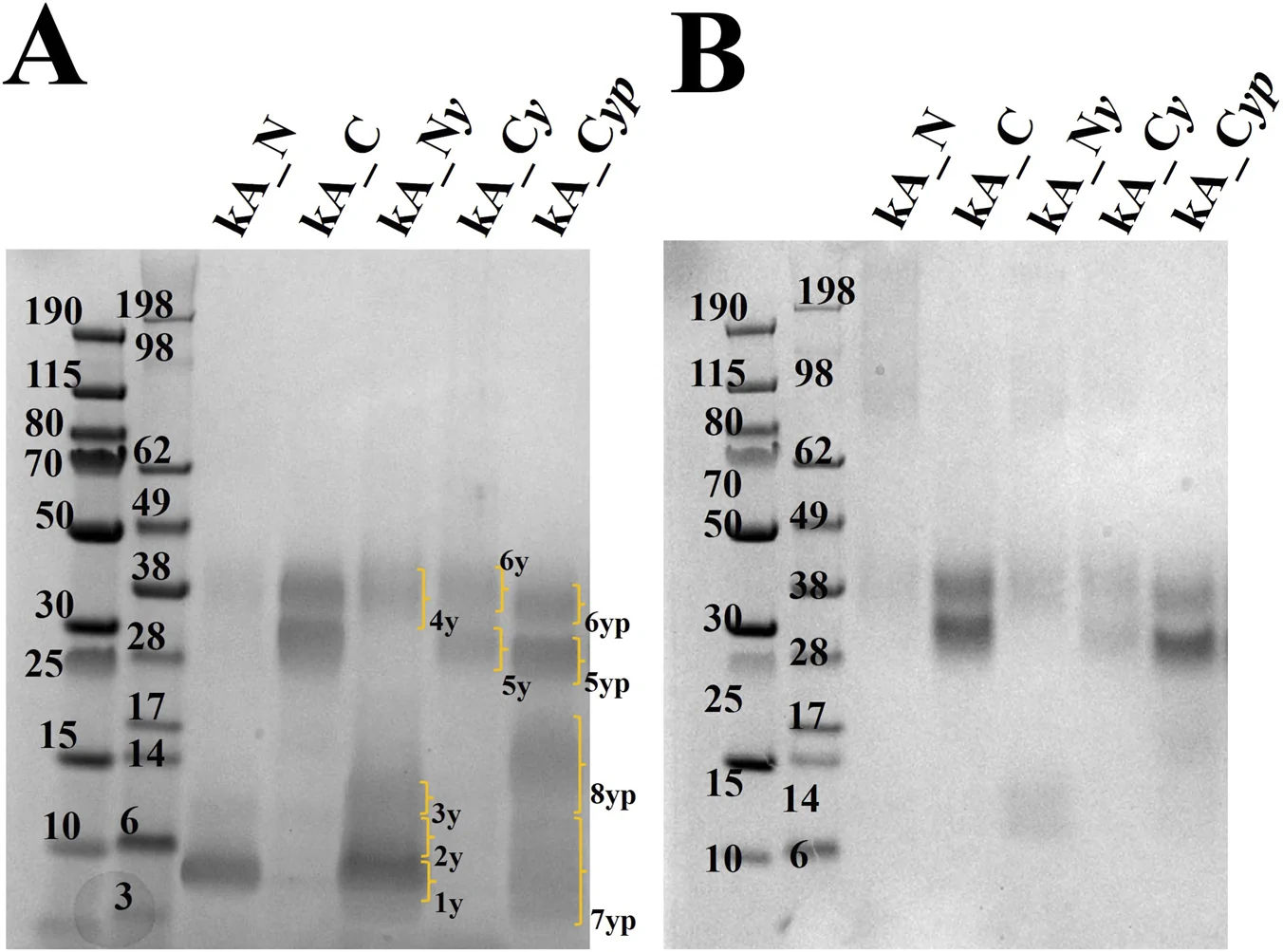
**(A)** Coomassie-stained SDS-PAGE (4%–12% acrylamide) of proteins purified from wild-type strains and protease knockouts by Ni-NTA chromatography. The amount of eluate loaded in each lane corresponds to ∼8 mL of cell-free culture broth, except for kA_Cyp (∼4 mL). Brackets indicate bands selected for in-gel digestion. **(B)** Glycoprotein (PAS) staining of a duplicate of the gel shown in panel **(A)**.

**FIGURE 7:**
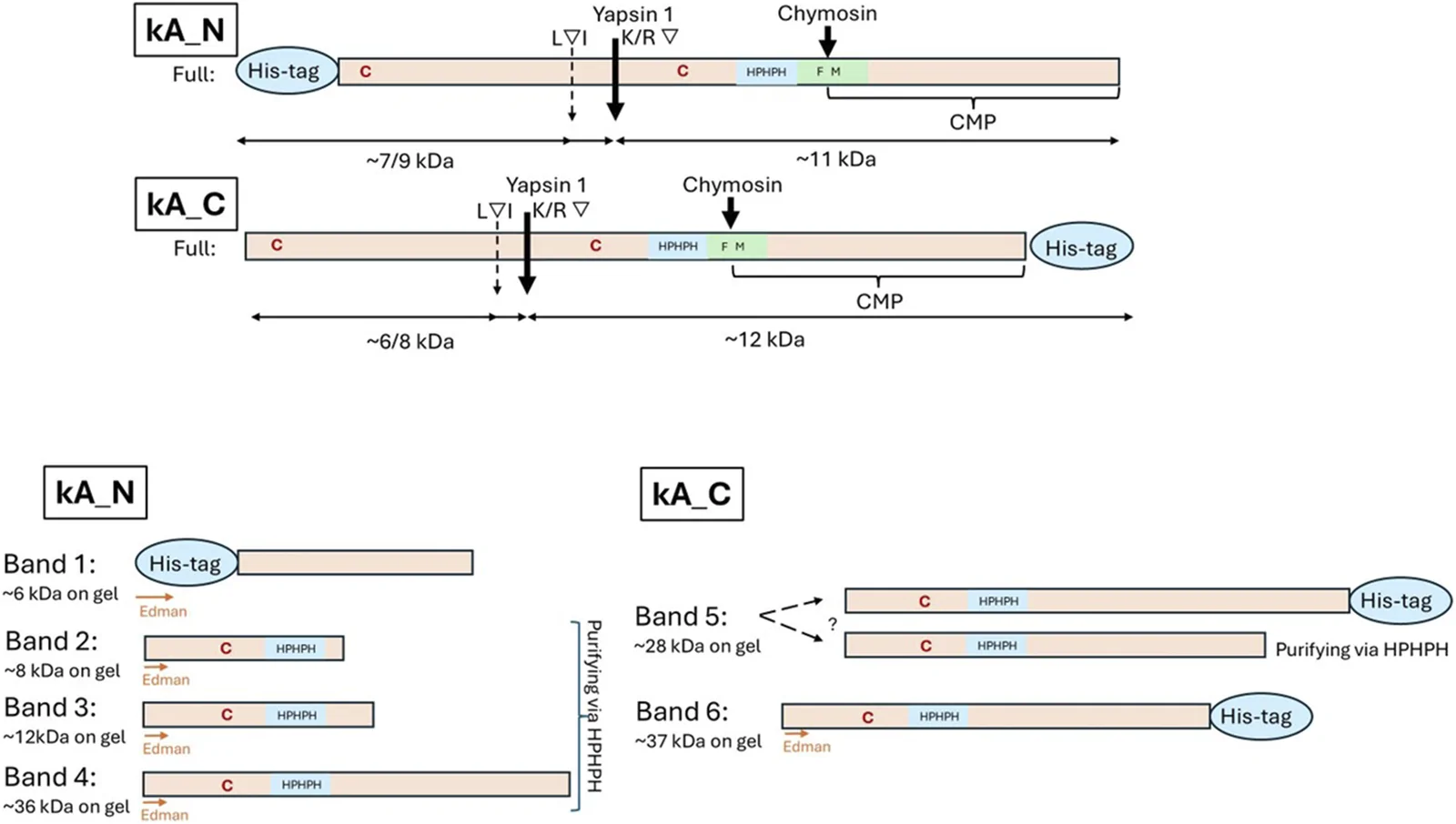
Schematic depiction of the full kA_N and kA_C molecules with indication of the chymosin cleavage site, the caseinomacropeptide (CMP) region, and the two cysteine residues that help stabilize native casein micelles (in red). Proteolytic (yapsin 1) cleavage sites identified by Edman sequencing in the wild-type strain are indicated by solid arrows, while the additional upstream cleavage site observed in the *yps1* knockout strain is indicated by a dashed arrow. The theorical molecular masses (excluding glycans) associated with the identified cleavage sites are indicated below the full-length protein. The major κ-casein-derived bands are shown below, together with their approximate apparent molecular weights in SDS-PAGE. The C-terminal ends of the fragments have not been identified so that the fragments without C-terminal His-tag may be shorter than illustrated.

A comparable band pattern in SDS-PAGE was also observed for the *pep4 yps1* double knockout strain, as two bands are still detected in kA_Cyp having similar electrophoretic mobility as the bands of kA_Cy (Figure 6A). However, this strain shows many additional low-molecular-weight bands (below ∼20 kDa), which are relatively intense. Therefore, although the Western blot (Figure 5) suggested a higher κ-casein yield in the *pep4 yps1* strain, it clearly does not perform better in terms of limiting proteolysis. It is well-known that *pep4* strains are not as robust as the *PEP4* wildtype and show a slower growth rate (Cereghino and Cregg, 2000). Indeed, although the fermentation of this strain took longer than that of the other strains (∼24 h additional induction time), the resulting biomass was roughly half that of the non-protease-disrupted strains (Supplementary Table S3). The seemingly higher production levels in the *pep4 yps1* strain may reflect a growth-production trade-off, where reduced allocation of cellular resources to growth can favor recombinant protein production. Alternatively, slower growth may alleviate secretion bottlenecks and thereby improve extracellular titers. As such, there may be room for improving titers by, e.g., optimization of feeding strategies. Still, assessing the impact of the protease deletions on productivity, rather than qualitative differences, was not a primary objective of this study, and the apparent differences in band intensity observed on the Western blot should not be overinterpreted. This caution is further reinforced by several confounding factors, including minor differences in parental background (X-33, GS115, and OPENPichia), undetermined copy number, and different integration loci (*his4* versus *AOX1*). Overall, the quantity of secreted κ-casein appeared to be fairly low, as the protein was only detectable by SDS-PAGE in the fermentation broth after Ni-NTA purification and concentration, or by Western blotting. FASP-LC-MS/MS of several of the strains presented in this study (kA, kA_N, kA_C, kA_Ny, kA_Cy) using a calibration curve generated with spiked ovalbumin as internal standards provided a rough estimate for bioreactor-produced single-copy κ-casein on the order of 10 mg/L (Supplementary Table S2). Interestingly, this is in a similar range as the single-copy yield obtained in shake-flasks (Section 3.1), while generally in *P. pastoris* productivity in bioreactors is much higher. This suggests κ-casein expression might impose inherent cell-physiological limitations.

### 3.4 Glycosylation

Given the smeared bands and much larger than expected molecular weights observed in SDS-PAGE and Western blot analyses, we assessed whether the protein is indeed glycosylated as hypothesized above. Based on the primary amino acid sequence of κ-casein, N-linked glycosylation can be excluded, as the protein lacks the consensus sequon for eukaryotic N-glycosylation (N-X-S/T, where X ≠ Pro; Marshall, 1974). However, *P. pastoris* is also capable of performing *O*-linked glycosylation, and κ-casein is *O*-glycosylated in cows. Glycoprotein detection was therefore done using periodic acid-Schiff (PAS) staining (Figure 6B). Most bands display PAS staining, indicating the presence of glycosylation, with the signal being particularly pronounced for higher-molecular-weight bands (between 28 and 38 kDa).

To further demonstrate that κ-casein is *O*-glycosylated, the protein was treated with Jack bean α(1–2,3,6)-mannosidase, an enzyme capable of removing *P. pastoris O*-linked mannose residues (Gomathinayagam and Hamilton, 2014). Following α-mannosidase treatment, the samples were analyzed by SDS–PAGE (Figure 8). The apparent molecular weight of recombinant κ-casein markedly decreased following treatment, confirming *O*-glycosylation. However, the bands remained smeared rather than becoming sharply defined. This may reflect partial proteolytic degradation, potentially due to contaminating proteases in the enzyme preparation, or incomplete removal of glycans.

**FIGURE 8:**
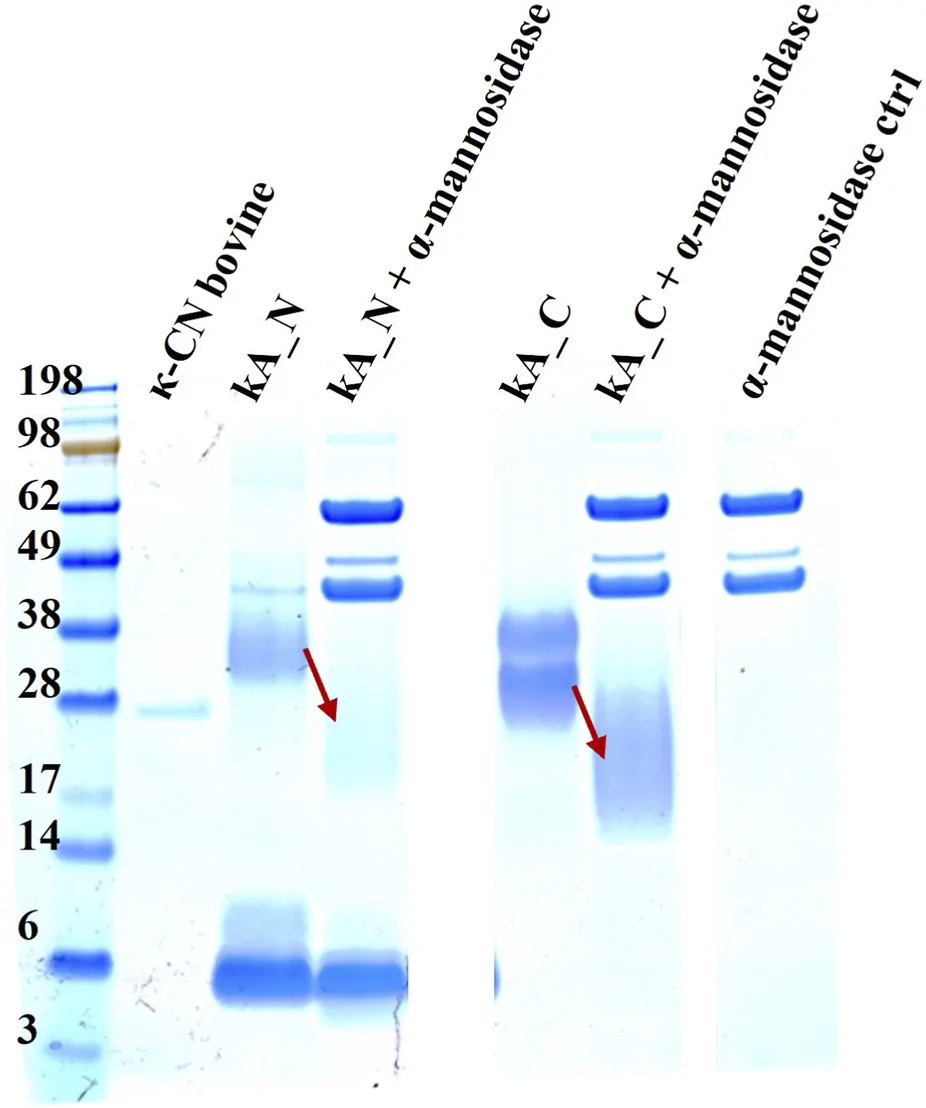
SDS-PAGE (4%–12% acrylamide) of untreated and Jack bean α-mannosidase-treated kA_N and kA_C samples. The enzyme alone is loaded for reference. Red arrows indicate the shift upon deglycosylation.

### 3.5 Mass spectrometry-based analyses

The bands of all N- and C-terminally His-tagged casein variants (Figures 3A, 6A) were analyzed by LC-MS/MS following in-gel digestion (IGD) for protein identification. To enable identification of non-tryptic peptides arising from host-derived proteolytic processing, spectra were additionally searched without assuming tryptic cleavage specificity. The observed bands were confirmed to be casein-derived as the identified peptides mapped to the construct sequence (Supplementary Figures S4,S5).

No peptide coverage was obtained for the C-terminal end of the protein, which constitutes a single large tryptic peptide of 53 amino acids. This is explained by the fact that, in MaxQuant, the software platform used for the identification of peptides from the mass spectrometry data, the maximum peptide length was set to 25 amino acids. The absence of internal peptides from this region suggests that no substantial *P. pastoris-*derived proteolytic cleavage occurred there, at least not to a degree that produces peptides falling within the detectable size range.

Peptides derived from the α-mating factor propeptide (EEGSVSLEKR and C-terminally truncated variants) were identified in several bands (2y, 3y, 4y and 6yp), indicating incomplete processing of the secretory leader. However, these peptides were generally present at low abundance (Supplementary Table S5), indicating that this applies only to a fraction of the molecules. EEGVSLEKR and shorter derivatives are common α-MF processing remnants (Keppler et al., 2026), caused by incomplete processing of the Kex2 site between the α-mating factor propeptide and the protein of interest.

The LC-MS/MS data were also inspected for possible phosphorylation of κ-casein. A targeted search was performed for peptide masses corresponding to phosphorylated serine, threonine, or tyrosine residues (+79.966 Da), but no phosphorylated κ-casein peptides were identified. This contrasts with the reported phosphorylation of β-casein produced in this host (Choi and Jiménez-Flores, 2001), although that protein was intracellularly retained.

Several attempts at MALDI-TOF mass spectrometry of different κ-casein samples were made. However, most spectra were of insufficient quality, likely due to the extensive glycosylation observed above in SDS-PAGE and PAS-staining (Figures 3A,6). A spectrum obtained for kA_C was of reasonable quality (Figure 9) and revealed a broad peak at ∼25 kDa consistent with such heavy glycosylation. Separate glycoforms could not be resolved for this highly heterogeneous sample, unlike an included mono-glycosylated bovine ribonuclease B control that did reveal the expected glycoforms (not shown).

**FIGURE 9:**
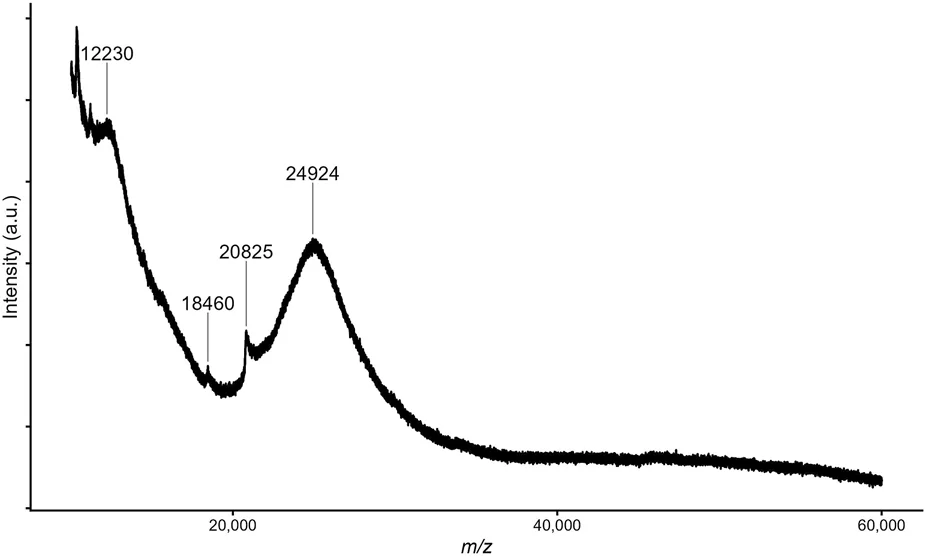
MALDI-TOF spectrum of Ni-NTA-purified kA_C.

### 3.6 Periplasmic retention

During the secretion process, some proteins may be efficiently translocated across the plasma membrane yet fail to traverse the cell wall, resulting in their accumulation within the periplasmic space. For that reason, the periplasmic fraction of several strains was analyzed by Western blotting. This revealed retention of κ-casein (Figure 10), although for some of the strains no clear signal was detected. This may reflect their overall lower expression levels, as the strains showing a detectable signal are those with higher extracellular titers according to Western blotting (Figures 4,5). Alternatively, this observation may indicate that periplasmic retention is more pronounced in high-producing strains or in physiologically stressed backgrounds, such as high-copy or *pep4 yps1* knock-out strains. As periplasmic retention has also been reported for β-casein in *Saccharomyces cerevisiae* (Jimenez-Flores et al., 1990), it may generally represent a research target for improving the extracellular yield of caseins.

**FIGURE 10:**
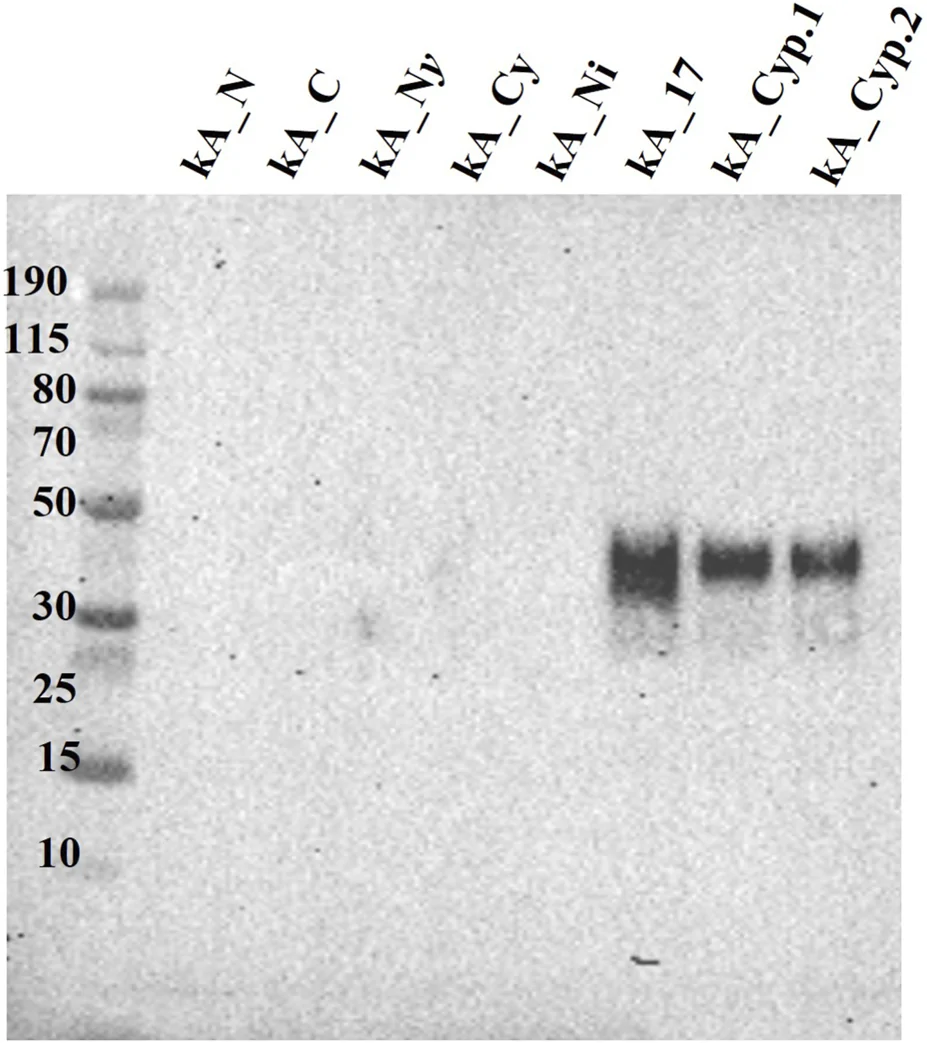
Western blot analysis (anti-κ-casein primary antibody) of periplasmic extracts from several bioreactor-grown strains presented in this study. Proteins were separated by SDS-PAGE (4%–12% acrylamide). Periplasmic proteins were isolated by enzymatic digestion of the cell wall with Zymolyase, and 19 µL of the resulting extract, corresponding to approximately 1.5 mg of wet cells, was loaded per lane. Strain kA_Ni is an intracellularly producing strain discussed in Section 3.7 and was included merely for completeness.

### 3.7 Intracellular expression

In addition to secretory expression, cytoplasmic expression of κ-casein was also investigated. For this purpose, the casein construct was cloned in the pPICZ A vector, which does not contain the α-MF secretory leader used in the other strains. The resulting strain (kA_Ni*)* was cultivated in a bioreactor as before, after which a cellular extract was prepared and analyzed by Western blotting. κ-Casein was detected as a major band migrating at ∼15 kDa using both anti-κ-casein and anti-His-tag primary antibodies (Figure 11). With the anti-His-tag antibody, additional lower-molecular-weight bands were observed. Together with the fact that 15 kDa is smaller than the expected ∼20 kDa molecular weight of intracellularly produced and therefore non-glycosylated κ-casein, this suggests the protein is degraded also when expressed intracellularly. The faint band at ∼50 kDa in anti-His-tag detection likely represents unspecific binding of the antibody. Considering the amount of cellular extract loaded for Western blot analysis with chemiluminescent detection, corresponding to ∼3.8 mg of wet cells, the observed signal suggests a low yield for intracellular expression.

**FIGURE 11:**
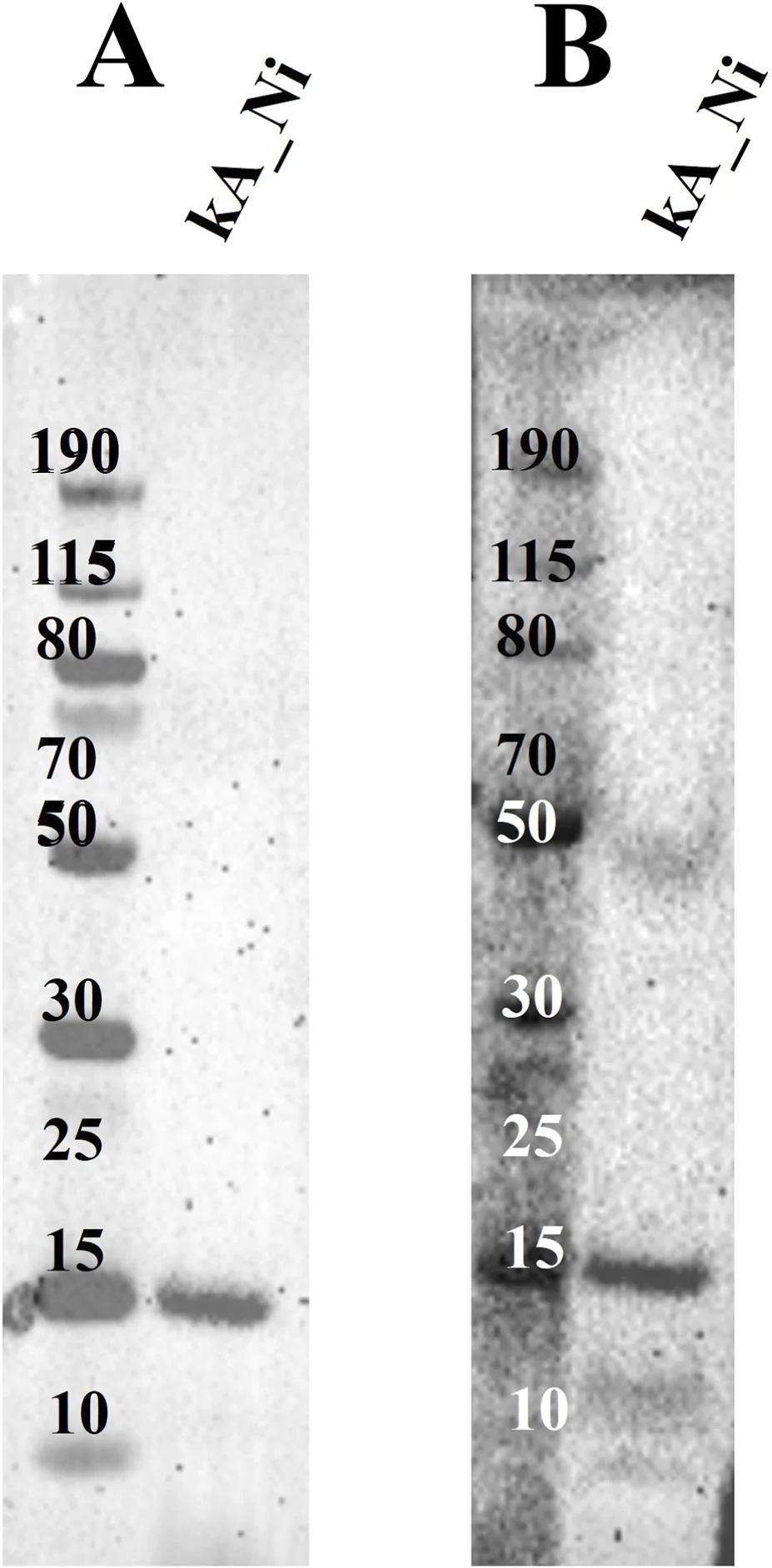
Western blot of κ-casein intracellularly expressed in a bioreactor. Proteins were separated by SDS-PAGE (4%–12% acrylamide). The amount of cell extract loaded corresponds to ∼3.8 mg wet weight per lane. **(A)** Detection with anti-κ-casein primary antibody. **(B)** Detection with anti-His-tag primary antibody.

## 4 Conclusion

Recombinant κ-casein was successfully produced and secreted, although titers in the cell-free broth of single-copy strains, both in shake flasks and bioreactors, were only in the tens of mg/L range. Increased gene copy number resulted in higher production levels; in shake flask cultures, production appeared to scale (sub-linearly) with copy number in the range tested.

Secreted κ-casein was extensively degraded. Although the use of protease knockout strains did not improve overall protein integrity, proteolytic cleavage patterns were affected. In a yapsin 1 knockout strain, the cleavage sites identified in the wildtype were no longer cleaved; however, an upstream proteolytic event emerged, indicating that multiple proteases were involved. A Protease A/yapsin 1 double knockout did not resolve cleavage of this secondary cleavage, and in fact resulted in additional low molecular weight bands.

The extensive proteolysis observed is likely related to the intrinsically disordered nature of κ-casein, which renders potential cleavage sites highly accessible to proteases. Secretory stress associated with recombinant protein production may contribute indirectly, for example, by affecting secretory or vacuolar protease activity. While the observed degradation contributes to the low levels of intact κ-casein recovered, other factors such as possible intracellular aggregation or self-assembly may also limit κ-casein productivity. The relative contribution of such mechanisms remains unclear and warrants further investigation.

The recombinant protein was extensively *O*-glycosylated, with no detectable phosphorylation. Periplasmic accumulation was observed, indicating that transit across the cell wall constitutes a bottleneck. Cytoplasmic expression showed low yield and still suffered from proteolytic degradation.

Future efforts to improve recombinant κ-casein production could target several of the limitations identified in this study. Identification of the proteases responsible for the remaining cleavage events would enable targeted strain engineering, while optimization of cultivation conditions, including temperature, pH, and medium composition, may further limit proteolysis. Alternative secretion leaders and protein fusion strategies could be explored to improve secretion efficiency. The observed periplasmic accumulation suggests that engineering of cell-wall properties may facilitate more efficient release of κ-casein into the extracellular medium.

The findings reported here highlight key challenges associated with the production of κ-casein in *Pichia pastoris*, and provide a basis for addressing them. This will be essential for improving product yield and integrity, thereby improving the feasibility of large-scale κ-casein production through precision fermentation.

## Data Availability

The datasets presented in this study can be found in online repositories. The names of the repository/repositories and accession number(s) can be found below: PRIDE, Project accession: PXD084422.
