# Supplementary material for: Precision fermentation of κ-casein using Pichia pastoris
Source: Front Bioeng Biotechnol. 2026 Oct 22;14:1946745. doi: 10.3389/fbioe.2026.1946745 (PMC13735683; doi:10.3389/fbioe.2026.1946745)
Supplement: Supplementary file 1 [file Supplementaryfile1.docx]

**SUPPLEMENTARY MATERIAL**

| **Construct** | **Protein Sequence** |
| --- | --- |
| kA, kA_7, kA_17 | QEQNQEQPIRCEKDERFFSDKIAKYIPIQYVLSRYPSYGLNYYQQKPVALINNQFLPYPYYAKPAAVRSPAQILQWQVLSNTVPAKSCQAQPTTMARHPHPHLSFMAIPPKKNQDKTEIPTINTIASGEPTSTPTTEAVESTVATLEDSPEVIESPPEINTVQVTSTAV |
| kA_N, kA_Ny | HHHHHHHHQEQNQEQPIRCEKDERFFSDKIAKYIPIQYVLSRYPSYGLNYYQQKPVALINNQFLPYPYYAKPAAVRSPAQILQWQVLSNTVPAKSCQAQPTTMARHPHPHLSFMAIPPKKNQDKTEIPTINTIASGEPTSTPTTEAVESTVATLEDSPEVIESPPEINTVQVTSTAV |
| kA_C, kA_Cy, kA_Cyp | QEQNQEQPIRCEKDERFFSDKIAKYIPIQYVLSRYPSYGLNYYQQKPVALINNQFLPYPYYAKPAAVRSPAQILQWQVLSNTVPAKSCQAQPTTMARHPHPHLSFMAIPPKKNQDKTEIPTINTIASGEPTSTPTTEAVESTVATLEDSPEVIESPPEINTVQVTSTAVGSHHHHHHHH |

**Table S1:** Amino acid sequences of the recombinant protein constructs used in this study

**A**

**B**

**Figure S1.** Relative quantification (RQ) values obtained by qPCR copy number analysis at the start and end of the cultivation process in (A) flasks and (B) bioreactor for clones kA_17 and kA_5, to assess copy number stability. The primers used in qPCR for detection of the expression cassette are specific to the *AOX1* promoter (Abad 2010). The start sample was collected at 0 h (immediately after inoculation), whereas the end sample was collected after ~48 h of methanol induction. Construct copy number (C) equals RQ-1, as the untransformed strain has a single endogenous copy of the *AOX1* promoter. Data are shown as the mean ± SD of three technical replicates.

**Figure S2.** Regions used for densitometric quantification in ImageJ.JS of the Western blot analysis of Fig. 1. The reference band of known protein amount (2 μg) is marked "R" and it was used to provide a rough estimate of the amount of the recombinant caseins based on relative band intensities.

**198**

**98**

**62**

**49**

**38**

**28**

**17**

**14**

**3**

**6**

**1**

**2**

**Figure S3.** SDS-PAGE analysis (10% acrylamide) visualized by Coomassie staining of kA_N purified under native conditions. Lane 1, purified in the absence of protease inhibitors. Lane 2, purified in the presence of protease inhibitors.

| Sample | iBAQ | | mg/L |
| --- | --- | --- | --- |
| kA | 1381800000 | 22 | |
| kA_N | 797495800 | 13 | |
| kA_C | 958575100 | 13 | |
| kA_Ny | 910256000 | 14 | |
| kA_Cy | 675628000 | 8 | |

**Table S2:** Intensity-Based Absolute Quantification (iBAQ) FASP LC-MSMS values of κ-casein quantified in the cell-free fermentation broth of the listed strains with corresponding protein concentrations. Protein concentrations were estimated from iBAQ values using a calibration curve generated with spiked ovalbumin standards and processed through the FASP workflow. The calibration curve was performed directly in the analyzed samples, allowing conversion of iBAQ intensities into absolute κ-casein concentrations (mg/L). Values represent a single determination per strain

| Strain | Glycerol batch duration (h) | Methanol fed-batch duration (h) | Wet cell weight (g/L) |
| --- | --- | --- | --- |
| kA | 27.5 | 43.4 | 468 |
| kA_N | 31.6 | 63.7 | 403 |
| kA_C | 22.57 | 46.7 | 501 |
| kA_Ny | 23.1 | 44.1 | 442 |
| kA_Cy | 23.1 | 44 | 451 |
| kA_Cyp | 23 | 92 | 207 |
| kA_Ni | 23.51 | 46.8 | 408 |

**Table S3:** Fermentation parameters and final biomass concentrations for the recombinant κ-casein-producing strains. The glycerol batch and methanol fed-batch phases are connected by a transition/glycerol fed-batch phase (see Section 2.8). As methanol feeding is initiated 1h from the start of this transition phase, the total methanol feeding time exceeds the fed-batch duration by 3 h. Wet cell weight (g/L) was determined at the end of each fermentation.

| Band | Cycle 1 | Cycle 2 | Cycle 3 | Cycle 4 | Cycle 5 | Cycle 6 | Cycle 7 | Sequence match |
| --- | --- | --- | --- | --- | --- | --- | --- | --- |
| 1 | E, G | A | H | G, (H) | X, R? | H, G | H | EAHHHHH |
| 2 | V, P, G, (S) | A, Q, L | A, Q, S | V, P, Q | R, I, A | S, L, G | P, I, (Q) | PAAVRSP  SPAQILQ |
| 3 | P, V | A | X, A, Q | V | R | S | P | PAAVRSP |
| 4 | G, S, V, P | A, P, Q | A, V | V, Q, E | R, I | L, S | P, G | PAAVRSP SPAQILQ |
| 6 | P, V, (S) | A, Q | A, V | V, Q | R, I | S, L | P, Q | PAAVRSP SPAQILQ |
| 6y | V, G, I | N, G, Q | G, N | Q, L | F, A | L, V | - | INNQFL |

**Table S4:** N-terminal Edman sequencing results for each band with calls for each cycle made by service provider expert. Due to the extensive proteolytic degradation of κ-casein and resulting closely spaced bands, mixed reactions were obtained as expected. Residues between parentheses indicate low level signals. Underlined residues do not (fully) match the call in that cycle.

**Figure S4:** Mapping of peptides identified by LC-MS/MS following in-gel digestion of selected bands (see Figures 4A and 6A). Comparison of N-terminally His-tagged κ-casein SDS-PAGE bands in wild-type and protease knockout strains (y: *yps1* strain).

**Figure S5:** Mapping of peptides identified by LC-MS/MS following in-gel digestion of selected bands (Figures 4A and 6A). Comparison of C-terminally His-tagged κ-casein SDS-PAGE bands in wild-type and protease knockout strains (y: yps1 strain, yp: yps1 pep4 strain).

| **Raw intensity** | | | | | | |
| --- | --- | --- | --- | --- | --- | --- |
| Peptide | | Band 2y | Band 3y | | Band 4y | Band 6yp |
| EEGVSLE | - | | | - | - | 644680 |
| EEGVSLEK | | 3818100 | 673170 | | - | 882820 |
| EEGVSLEKR | | 1685800 | 188830 | | 2212100 | 703480 |
|  | |  |  | |  |  |
| Total | | 11680800 | 5462392 | | 40555000 | 2283339720 |
|  | |  |  | |  |  |
| **Relative abundance (%)** | | | | | | |
| Peptide | | Band 2y | Band 3y | | Band 4y | Band 6yp |
| EEGVSLE | | - | - | | - | 0.03 |
| EEGVSLEK | | 32.69 | 12.32 | | - | 0.04 |
| EEGVSLEKR | | 14.43 | 3.46 | | 5.45 | 0.03 |

**Table S5**: Raw intensities and relative abundances of α-mating factor -derived peptides identified by LC–MSMS analysis after IGD for kA_C in *yps1* and *yps1 pep4* strains. Relative abundance (%) was calculated as the intensity of a given peptide divided by the total intensity of all κ-casein-derived peptides identified in the corresponding band

X

X

X

X

**Figure S6.** Original Western blot corresponding to Fig. 1. Lanes marked with an X contain samples unrelated to the present analysis.

**Figure S7.** Original Western blot corresponding to Fig. 2A. Exposure time: 300 seconds

**Figure S8.** Original Western blot corresponding to Fig. 2B. Exposure time: 133 seconds

**Figure S9.** Original Western blot corresponding to Fig. 3. Exposure time: 101 seconds

X

**Figure S10.** Uncropped Coomassie-stained SDS-PAGE gels corresponding to Fig. 3. Lanes marked with an X contain samples unrelated to the present analysis.

**Figure S11.** Original Western blot corresponding to Fig. 5. Exposure time 93 sec.

X

X

**Figure S12.** Original Coomassie-stained SDS-PAGE gel corresponding to Fig. 8. Lanes marked with an X contain samples unrelated to the present analysis.

**Figure S13.** Original Western blot corresponding to Fig. 9. Exposure time 500 sec.

**Figure S14.** Original Western blot corresponding to Fig. 10A. Exposure time: 600 seconds

**Figure S15.** Original Western blot corresponding to Fig. 10B. Exposure time: 460 seconds
